# Rubella virus genotype 2B endemicity and related utility of serum-based molecular characterization in Uganda

**DOI:** 10.1186/s13104-023-06499-5

**Published:** 2023-09-14

**Authors:** Phionah Tushabe, Barnabas Bakamutumaho, James Peter Eliku, Molly Birungi, Francis Aine, Prossy Namuwulya, Henry Bukenya, Immaculate Ampeire, Annet Kisakye, Charles R. Byabamazima, Josephine Bwogi

**Affiliations:** 1 EPI-Laboratory, Uganda Virus Research Institute, P.O. Box 49, Entebbe, Uganda; 2 World Health Organization, Uganda Country Office, P.O. Box 24578, Kampala, Uganda; 3 Ministry of Health Uganda, P.O. Box 7272, Kampala, Uganda; 4 WHO Inter-Country Support Team Office for Eastern and Southern Africa (IST/ESA), Harare, Zimbabwe

**Keywords:** Rubella, Congenital Rubella Syndrome, Genotypes, Endemic, Sera

## Abstract

There are 13 globally recognized rubella virus genotypes of which only 2 (1E and 2B) have been detected recently. The largest percentage of all reported rubella virus sequences come from China and Japan with Africa reporting limited data. In a bid to address the lack of rubella genotype data in Uganda and the World Health Organization Africa region, we sought to characterize rubella viruses retrospectively using sera collected from suspected measles patients that turned out rubella IgM positive.

Seven sequences belonging to genotype 2B sub-lineage 2B-L2c were obtained. These sequences clustered with other genotype 2B sequences previously reported from Uganda. None of the other genotypes (1E and 1G) reported from Uganda in the earlier years were detected. In addition, none of the sequences were obtained after the introduction of the measles-rubella containing vaccine. The above highlight the need for continuous rubella virological surveillance to confirm interruption of endemic rubella genotype circulation.

## Introduction

The rubella virus, a member of Genus *Rubivirus* in the family *Matonaviridae*, is a positive-sense single stranded RNA virus that causes mild disease among humans. Although humans are the only known reservoir, the rubella virus has recently been shown to be related to the *Rubiviruses* Ruhugu and Rustrela, found in bats (*Hipposideros cyclops*) and mice (*Apodemus flavicollis*) respectively [1], suggesting a potential zoonotic origin. Rubella virus infection in the first trimester of pregnancy has teratogenic effects on the unborn foetus that result into congenital rubella syndrome (CRS) among children, for which vaccination is a major component of the initiatives to its elimination and the associated disease burden. The available reports of 2010 indicate high CRS incidences of 116 and 121 per 100,000 live births in Africa and South East Asia respectively [2], representing the highest global burden at the time.

In Africa, rubella surveillance is leveraged on measles case-based surveillance and utilizes sera that tests negative for active measles infection while CRS sentinel surveillance was initiated in selected countries (including Uganda) between 2011 and 2016 [3]. Because of the above, rubella molecular surveillance is not comprehensive resulting into under reporting of rubella virus sequence data. Even when rubella virus circulation is intense as was the case in 2018 across the Africa and Eastern Mediterranean WHO regions, there is often no reported rubella genotype data [4], presenting missed opportunities to document circulation of associated strain(s) and further highlighting gaps in molecular surveillance. Rubella virus genotype data is grossly lacking in Uganda and generally in the WHO Africa region and this is partly attributed to the limitations associated with the scarcity and timing of collection of the preferred specimens (that is throat swabs and oral fluids) for molecular characterization.

We previously reported on the utility of sera as an alternative specimen for molecular characterization of rubella viruses [5] through collaborative work that was conducted at the US Centres for Disease Control and Prevention in Atlanta, Georgia. Here in, we report on rubella genotype endemicity using the serum-based technique that we established at the Uganda Virus Research Institute.

## Main text

### Methods

From January 2019 to April 2022, serum specimens collected from suspected measles patients through the measles case-based surveillance programme were tested for both measles and rubella IgM at Uganda Virus Research Institute. Different commercial enzyme-linked immunosorbent (ELISA) kits were used; the Enzygnost® Anti-Measles Virus/IgM and Enzygnost® Anti-Rubella Virus/IgM kits (Siemens, Marburg, Germany) in 2019 and the Anti-Measles Virus ELISA and the Anti-Rubella Virus Glycoprotein ELISA IgM kits (Euroimmun, Lübeck, Germany) from 2020 to 2022. The measles IgM negative but rubella IgM positive sera collected within two days of rash onset, with volumes greater than 300 µl, were selected for molecular characterization. RNA extraction and genotyping assays were done as described previously [5]. The resulting DNA was purified using the Invitrogen ChargeSwitch™ PCR Clean-Up kit (Life Technologies Corp, Carlsbad, CA, USA) following the manufacturer’s instructions then sequenced bidirectionally using the Applied Biosystems BigDye™ Terminator v3.1 Cycle Sequencing kit (Thermo Fisher Scientific, Vilnius Lithuania). The sequencing reaction products were cleaned using the Agencourt CleanSEQ kit (Beckman Coulter Inc, CA, USA) following the manufacturer’s guidelines and loaded onto an ABI 3500 genetic analyzer (Applied Biosystems).

The 739-nt sequence windows required for rubella genotype determination were obtained following analysis using Geneious prime version 2022.0.2 (Biomatters Ltd) and genotypes determined as previously described [5]. The sequences were deposited into GenBank with the accession numbers ON861808 – ON861814. These sequences together with genotype 2B sub-lineage sequences [6] and 2B sequences from the Africa region that were available in GenBank as of June 27, 2022 were aligned using MAFFT [7] and a Maximum-likelihood phylogenetic tree based on the Tamura-Nei plus empirical base frequencies plus gamma model generated using IQ-TREE [8] and run for 1000 pseudo replicates. The tree was visualized using the Interactive Tree Of Life (iTOL) [9] and bootstrap values greater than 75% are shown at the nodes.

## Results

**Fig. 1:**
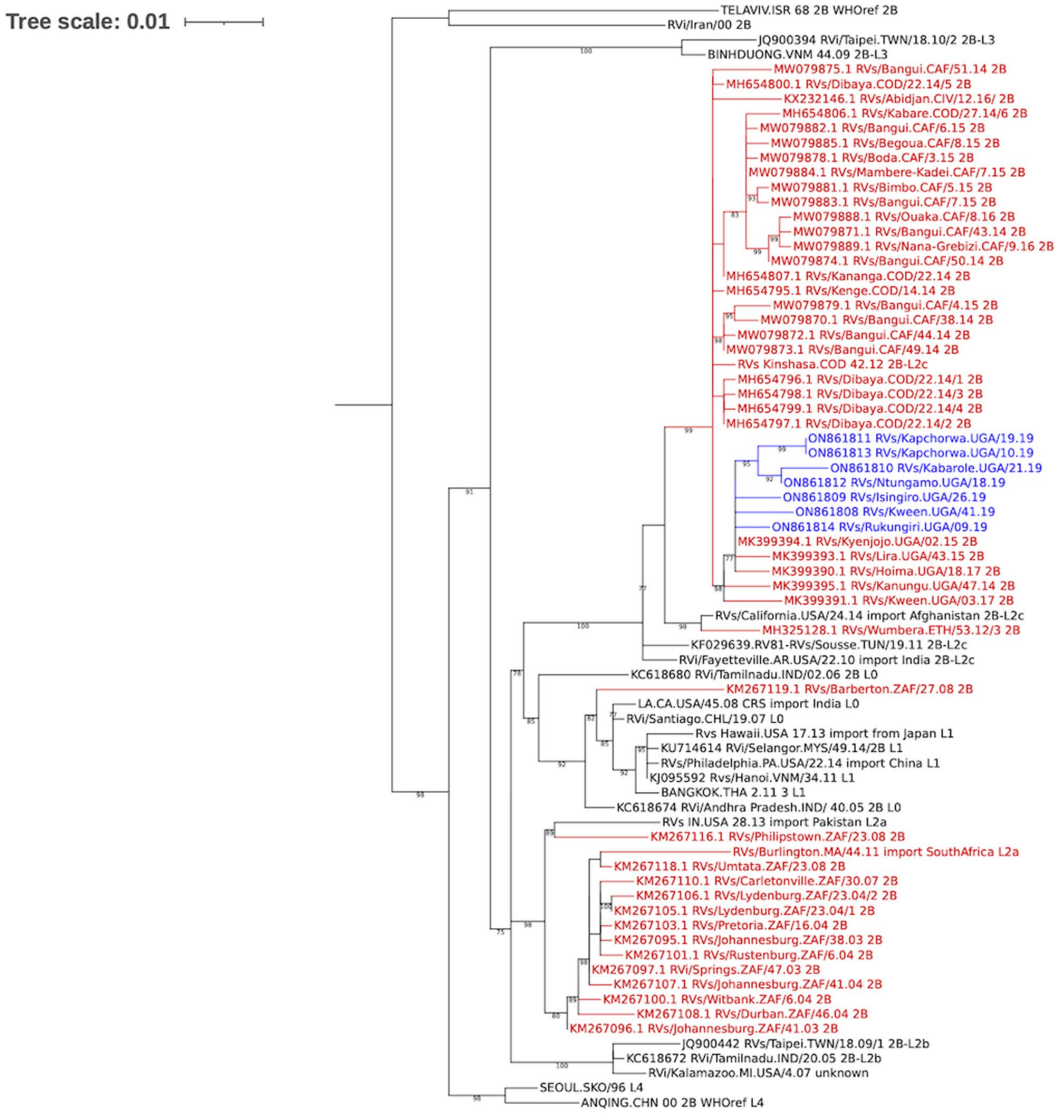
Maximum-likelihood phylogenetic tree comparing study sequences with 2B sub-lineage sequences and sequences from Africa region Legend: Blue sequences – sequences obtained from this study; Red sequences – sequences from the Africa region; Black sequences – rubella genotype 2B sub-lineage sequences The sub-lineage sequences and the other genotype 2B sequences were downloaded from GenBank

A total of 2,974 sera were received from suspected measles patients through the measles case-based surveillance programme. Of these, 337 (11.3%) were rubella IgM positive and negative for measles IgM (Table 1 summarises the demograghics of these patients). One hundred and fifty-three (45.4%) had been collected within 2 days of rash onset and thus were eligible for genotyping. RNA was extracted from 75 (49%) samples with adequate volumes and 15 (20%) of these were real-time RT-PCR positive. Seven (46.7%) of the 15 samples were sequenced successfully and the 739-nt window of the E1 glycoprotein obtained. All sequences belonged to genotype 2B sub-lineage 2B-L2c (Fig. 1) and were obtained from sera collected in 2019. The 7 Ugandan 2B sequences clustered with other previously reported Ugandan genotype 2B sequences collected in 2014, 2015 and 2017 as well as sequences from Africa except for South Africa (Fig. 1).

**Table 1:** Summary of the distribution of rubella cases by year, region and agegroup

Region	Year	< 5years	5-<10 years	10-<15 years	> 15 years	TOTAL
Central	2019	17	13	4	3	**37**
	2020	3	2	0	0	**5**
	2021	9	3	0	0	**12**
	2022	1	0	0	0	**1**
	**TOTAL**	**30**	**18**	**4**	**3**	**55**
Eastern	2019	26	23	3	0	**52**
	2020	1	0	0	0	**1**
	2021	6	1	1	0	**8**
	2022	1	0	0	0	**1**
	**TOTAL**	**34**	**24**	**4**	**0**	**62**
Northern	2019	42	27	7	3	**79**
	2020	0	0	0	0	**0**
	2021	4	2	0	0	**6**
	2022	2	0	0	0	**2**
	**TOTAL**	**48**	**29**	**7**	**3**	**87**
Western	2019	55	55	9	5	**124**
	2020	1	0	0	0	**1**
	2021	7	1	0	0	**8**
	2022	0	0	0	0	**0**
	**TOTAL**	**63**	**56**	**9**	**5**	**133**

Three of the 7 sequences were from the Eastern region while the remaining sequences were from the Western region of the country. Kapchorwa district (Eastern region) had two cases that occurred within two months of each other, from which sequences were obtained (Table 2). The sequence from Kween district (Eastern region) was not similar to the ones from Kapchorwa district signifying an independent occurrence 22 weeks after (Fig. 1). The sequences from the districts in the Western region though obtained within a period of 17 weeks were not similar (Fig. 1).

**Table 2:** Summary of demographics and geographic distribution of the patients from whom sequences were obtained

WHO name	Accession number	District of origin	Age (months)
RVs/Kween.UGA/41.19	ON861808	Kween	72
RVs/Isingiro.UGA/26.19	ON861809	Isingiro	58
RVs/Kabarole.UGA/21.19	ON861810	Kabarole	25
RVs/Kapchorwa.UGA/19.19	ON861811	Kapchorwa	48
RVs/Ntungamo.UGA/18.19	ON861812	Ntungamo	72
RVs/Kapchorwa.UGA/10.19	ON861813	Kapchorwa	36
RVs/Rukungiri.UGA/09.19	ON861814	Rukungiri	156

We retrieved a total of 1,261 genotype 2B sequences from GenBank and of these, 581 unique sequences were retained for analysis. Approximately 67% of all the sequences reported were from the Western Pacific region (WPRO) with the Africa region (AFRO) reporting only 44 (7.6%) sequences.

## Discussion

The sustained circulation of the rubella genotype 2B in the pre-vaccination era signifies established endemicity in Uganda. This is an important benchmark against which to monitor progress towards disease elimination. Uganda introduced a measles-rubella (MR) containing vaccine in October 2019 through a nationwide campaign that targeted children aged 9 months to under 15 years [10]. The country then switched to the MR vaccine administered at 9 months of age in the routine immunization schedule. Since then, rubella incidence proportions declined significantly by 12%, from approximately 22% in the prevaccination period to about 7% in the post-vaccination period within a short period of close to 2 years [11]. However, this may not be solely attributed to vaccination since in 2020, nationwide travel restrictions and redirection of resources in response to the COVID-19 pandemic adversely affected measles-rubella surveillance in the country and the entire Africa region [12]. To note, other African countries that implemented MR targeting the same agegroup prior to COVID-19 observed substantial reduction of up to 81% in the rubella incidence proportion [13].

First reported in Uganda in 2014 [5], rubella virus genotype 2B has also been reported in the Democratic Republic of Congo [14], Cote d’Ivoire [15], and Central African Republic [16] in Africa. It is unknown if this genotype circulated before its initial detection or if its emergence was as a result of importation(s), however, we did not observe clustering (Fig. 1) with other viruses from the region reemphasizing endemicity. The previously endemic genotypes 1E and 1G [5, 17] were not detected during this period suggesting that these could have been outcompeted by the circulating genotype 2B, which has been shown to have the widest geographical distribution of all the rubella genotypes [6]. However, it should be noted that this analysis considered only a small number of sequences and as such may not be representative of countrywide rubella circulation. And, although rubella is largely endemic in the Africa region, suboptimal virologic surveillance has not enabled elucidation of the complete genotype circulation scope in the region [6].

The two sequences from Kapchorwa district (Eastern region) were identical and possibly represent a contact transmission event since they were collected within two months of each other, and the rubella virus has an estimated overall substitution rate of 10^− 3^ subs/site/year [18]. The level of sequence identity and evidence of related transmissions in different regions of the country signify endemic transmission (Fig. 1). All the sequences belonged to sub-lineage 2B-L2c along with other previously reported sequences from Uganda and other African countries except for South Africa that clustered with those of sub-lineage 2B-L2a (Fig. 1). This highlights the need for classification up to sub-lineage level during rubella molecular surveillance in order to characterize accurately the genetic diversity of circulating strains and ultimately, aid in tracking transmission patterns more effectively.

Sub-Saharan Africa (SSA) shoulders 25% of the overall all-cause global disease burden [19] but only 1.4% of published data is known on associated factors as compared to for example 20.2% from China alone [20]. This highlights a global imbalance in information sharing which is also reflected in rubella molecular surveillance with approximately 67% of genotype 2B sequences reported from the Western Pacific region while the Africa region reported only 7.6%. The reported sequences were from only 6 of its 47 countries that is Ethiopia, Democratic Republic of Congo, Cote d’Ivoire, Central African Republic, South Africa, and Uganda. Rubella molecular surveillance data is unavailable from 41 countries in the Africa region where the disease incidence rate is persistently higher than the global estimates, ranging from 4.2 to 11 per 1,000,000 population between 2014 and 2021 [21]. With such high disease burden, immunization programs need to be enhanced and molecular surveillance emphasized to assess the efficacy of these programs. The routine measles-rubella surveillance offers an opportunity to advance related molecular surveillance through application of serum-based genomic sequencing. And, although these viruses were from a limited catchment area, serum based genomic surveillance offers a potential option for continuous virological surveillance to monitor for possible interruption of endemic rubella genotypes in circulation.

## Limitations

- We recovered few rubella sequences because archived sera, through multiple freeze-thaw cycles, might have suffered viral RNA degradation. It will hence be important to utilize this serum-based approach in real time specimens after collection.
- Since we relied on unvalidated historical data, we could have included sera collected later than two days from illness onset resulting into low recovery of the viral genomes. Hence, late detection of cases outside of the eligible inclusion period post onset could have eliminated opportunities of candidates for genotype(s) detection.
- We did not have sequence data for 2020 and 2021 which represents a molecular surveillance gap. This could be due to the COVID-19 pandemic, however, it may not undermine our findings given the sufficient genomic data (Fig. 1).

## Data Availability

All the data generated during the current study are included in this manuscript. The sequences obtained in this study are available in GenBank under the accession numbers ON861808 – ON861814.

## List of abbreviations

AFRO: Africa region
COVID-19: Coronavirus disease 2019
CRS: Congenital Rubella Syndrome
DNA: Deoxyribonucleic acid
ELISA: Enyzme linked immunosorbent assay
iTOL: Interactive Tree Of Life
MR: measles-rubella
PCR: polymerase chain reaction
RNA: Ribonucleic acid
SSA: Sub-Saharan Africa
UGA: Uganda
WHO: World Health Organisation
WPRO: Western Pacific Region

## References

[1] Bennett AJ, Paskey AC, Ebinger A, Pfaff F, Priemer G, Höper D (2020). Relatives of rubella virus in diverse mammals. Nature.

[2] Vynnycky E, Adams EJ, Cutts FT, Reef SE, Navar AM, Simons E et al. Using seroprevalence and immunisation coverage data to estimate the global burden of Congenital Rubella Syndrome, 1996–2010: A systematic review [Internet]. Vol. 11, PLoS ONE. 2016. p. e0149160. 10.1371/journal.pone.0149160. 10.1371/journal.pone.0149160 PMC4786291 26962867

[3] Masresha B, Shibeshi M, Kaiser R, Luce R, Katsande R, Mihigo R (2018). Congenital Rubella Syndrome in the African Region - Data from Sentinel Surveillance. J Immunol Sci.

[4] Amy KW, William JM, Rubella (2022). Lancet.

[5] Tushabe P, Bwogi J, Abernathy E, Birungi M, Eliku JP, Seguya R (2020). Descriptive epidemiology of rubella disease and associated virus strains in Uganda. J Med Virol.

[6] Rivailler P, Abernathy E, Icenogle J (2017). Genetic diversity of currently circulating rubella viruses: a need to define more precise viral groups. J Gen Virol.

[7] Madeira F, Park YM, Lee J, Buso N, Gur T, Madhusoodanan N (2019). The EMBL-EBI search and sequence analysis tools APIs in 2019. Nucleic Acids Res.

[8] Nguyen LT, Schmidt HA, Von Haeseler A, Minh BQ (2015). IQ-TREE: a fast and effective stochastic algorithm for estimating maximum-likelihood phylogenies. Mol Biol Evol.

[9] Letunic I, Bork P (2021). Interactive tree of life (iTOL) v5: an online tool for phylogenetic tree display and annotation. Nucleic Acids Res.

[10] World Health Organization Uganda. Uganda scores highly in the Measles-Rubella-Polio vaccination campaign. 2019.

[11] Mensah AE, Gyasi OS (2022). Measles-Rubella Positivity Rate and Associated factors in Pre-Mass and Post-Mass Vaccination Periods: analysis of Uganda Routine Surveillance Laboratory Data. Adv Public Heal.

[12] Masresha B, Luce R, Katsande R, Dosseh A, Tanifum P, Lebo E (2021). The impact of the covid-19 pandemic on measles surveillance in the world health organisation african region, 2020. Pan Afr Med J.

[13] Luce R, Masresha B, Katsande R, Fall A, Shibeshi M (2018). The impact of recent rubella vaccine introduction in 5 countries in the African Region. J Immunol Sci.

[14] Pukuta E, Waku-Kouomou D, Abernathy E, Illunga BK, Obama R, Mondonge V (2016). Genotypes of rubella virus and the epidemiology of rubella infections in the Democratic Republic of the Congo, 2004–2013. J Med Virol.

[15] Kadjo HA, Waku-Kouomou D, Adagba M, Abernathy ES, Abdoulaye O, Adjogoua E (2018). Epidemiology of rubella infection and genotyping of rubella virus in Cote d’Ivoire, 2012–2016. J Med Virol.

[16] PAGONENDJI MS, GOUANDJIKA-VASILACHE I, CHARPENTIER E, SAUSY A, LE FAOU A, DUVAL RE (2021). Rubella epidemiology in the central African Republic, 2015–2016 and molecular characterization of virus strains from 2008–2016. Int J Infect Dis.

[17] Namuwulya P, Abernathy E, Bukenya H, Bwogi J, Tushabe P, Birungi M (2014). Phylogenetic analysis of rubella viruses identified in Uganda, 2003–2012. J Med Virol.

[18] Padhi A, Ma L. Molecular evolutionary and epidemiological dynamics of genotypes 1G and 2B of rubella virus. PLoS One [Internet]. 2014;9(10):e110082. 10.1371/journal.pone.0110082. 10.1371/journal.pone.0110082 PMC4201520 25329480

[19] Byrne D. Science in Africa: a continent on the cusp of change [Internet]. 2022 [cited 2022 Jul 15]. Available from: https://www.nature.com/articles/d41586-022-01147-7. 10.1038/d41586-022-01147-7 35478022

[20] United Nations Educational Scientific and Cultural, Organization. UNESCO Global Science Report: towards 2030. UNESCO Global Science Report: towards 2030; 2015.

[21] World Health Organization. Rubella reported cases and incidence [Internet]. 2022 [cited 2022 Jul 15]. Available from: https://immunizationdata.who.int/pages/incidence/rubella.html.

